# Extracellular Vesicles Derived from Adipose Tissue Macrophages in Obesity: Functional Heterogeneity and miRNA-Mediated Regulation of Skeletal Muscle Insulin Sensitivity

**DOI:** 10.3390/biology15212039

**Published:** 2026-11-08

**Authors:** Menglan Li, Ling Wang, Yiwen Geng, Zhuochang Feng, Da Liu, Yongzhi Deng

**Affiliations:** 1 School of Acupuncture and Tuina, Changchun University of Chinese Medicine, Changchun 130117, China; lml981118@163.com; 2 School of Traditional Chinese Medicine, Changchun University of Chinese Medicine, Changchun 130117, China; wl787352570@163.com (L.W.); fengchangzhuo@ccucm.edu.cn (Z.F.); 3 School of Pharmacy, Changchun University of Chinese Medicine, Changchun 130117, China; gengyw6135@ccucm.edu.cn; 4 Public Experimental Center, Changchun University of Chinese Medicine, Changchun 130117, China

**Keywords:** obesity, adipose tissue macrophages, extracellular vesicles, microRNA, skeletal muscle, insulin sensitivity

## Abstract

Remodeling of adipose tissue macrophages (ATMs) during obesity is closely linked to insulin resistance. In addition to soluble mediators, extracellular vesicles (EVs) released by ATMs and their microRNA (miRNA) cargo have been implicated in metabolic communication between adipose tissue and skeletal muscle. Previous studies have shown that EV cargo profiles and metabolic effects can vary across ATM states, adipose depots, and macrophage populations, with some EVs promoting insulin resistance and others improving insulin sensitivity. Among the mechanisms relevant to skeletal muscle, the miR-29a–PPARδ relationship has relatively direct experimental support from ATM-EV studies. The miR-210-3p–GLUT4 relationship is supported by changes in ATM-EV cargo, findings from myocytes and other recipient-cell models, and experiments involving exogenously administered macrophage EVs. However, this relationship has not been fully validated within a single ATM-EV–skeletal muscle experimental system. Studies involving miR-690 also suggest that EVs from certain macrophage populations can improve insulin sensitivity, although the molecular annotation of miR-690, its reported direct targeting mechanism, and the applicability of these findings to humans remain under debate. Overall, these findings implicate ATM-derived EVs in metabolic regulation, while their physiological effects on skeletal muscle in humans remain uncertain.

## Simple Summary

Obesity changes the immune environment of fat tissue and can reduce the ability of skeletal muscle to respond to insulin, a hormone that helps regulate blood sugar. Macrophages, a type of immune cell found in fat tissue, release tiny membrane-enclosed particles that carry biological signals to other cells. Studies in animals and cultured cells suggest that these particles can influence insulin sensitivity, but their effects vary depending on the macrophages that release them. Some particles are associated with impaired glucose uptake, whereas others appear to improve it. Small RNA molecules carried by these particles may contribute to these differences. This review examines how macrophages change during obesity, how their released particles affect metabolism, and what is known about their effects on skeletal muscle. Although laboratory studies have identified several possible mechanisms, it remains uncertain how extensively this communication occurs naturally in the body or contributes to insulin resistance in humans. Understanding these relationships may provide new insights into obesity-related metabolic disorders.

## 1. Introduction

Obesity-associated chronic low-grade inflammation is an important contributor to the development and progression of insulin resistance (IR) [1,2,3,4]. Adipose tissue macrophages (ATMs) play a major role in obesity-associated adipose tissue inflammation and undergo substantial remodeling during this process [5,6]. Skeletal muscle is the primary peripheral tissue responsible for insulin-mediated glucose uptake and utilization, accounting for approximately 80% of whole-body insulin-stimulated glucose disposal [7]. Understanding how changes in ATMs within obese adipose tissue affect skeletal muscle insulin sensitivity is therefore important for elucidating systemic IR.

Previous research on metabolic communication between adipose tissue and skeletal muscle has largely focused on soluble mediators, including cytokines and adipokines. In recent years, extracellular vesicles (EVs) have emerged as important carriers of signals between metabolic tissues [8,9,10,11]. Experimental studies have shown that EVs derived from obese ATMs and their microRNA (miRNA) cargo can affect insulin-responsive cells, including skeletal muscle cells, altering glucose metabolism and insulin sensitivity [12,13,14]. EVs derived from macrophages in different activation states, adipose depots, and ATM populations have also been reported to exert distinct metabolic effects [15,16,17], suggesting that donor cell characteristics may influence EV-mediated metabolic regulation.

Previous reviews have covered macrophage-derived EVs, adipose tissue-derived EVs, and diabetes-associated EV miRNAs [8,9,10,11,18]. This review examines how ATM state and adipose depot relate to EV-associated metabolic effects, focusing on evidence connecting these signals to skeletal muscle insulin sensitivity. It distinguishes direct ATM-EV findings from results obtained with other macrophage sources, independent recipient-cell models, or human association studies, and evaluates whether the available data support endogenous ATM-to-skeletal-muscle communication under physiological conditions.

## 2. Obesity-Associated ATM Remodeling and Changes in EV-miRNA Signaling

### 2.1. Adipose Tissue Inflammation and ATM Remodeling in Obesity

Obesity-associated adipose tissue expansion is accompanied by immune cell recruitment, local inflammation, and marked accumulation of ATMs [5,6]. Early studies characterized ATMs as predominantly M2-like under homeostatic conditions, whereas proinflammatory features become more pronounced in obesity [6].

Lipid overload is an important contributor to this process. Elevated free fatty acids and associated metabolic stress in obesity can activate Toll-like receptor (TLR) signaling and affect inflammatory pathways, including c-Jun N-terminal kinase (JNK) and nuclear factor κB (NF-κB), promoting proinflammatory cytokine expression and local immunometabolic dysregulation [19,20,21,22]. Obesity-associated ATM remodeling therefore involves not only an increase in macrophage numbers but also changes in their inflammatory states and metabolic functions. The M1/M2 framework remains useful for describing simplified experimental macrophage activation states, but it does not capture the transcriptional, metabolic, and functional heterogeneity of ATMs in vivo.

### 2.2. ATM Heterogeneity and Adipose Depot Characteristics

Single-cell sequencing and transcriptomic studies have further revealed the heterogeneity of ATM populations in obese adipose tissue. Among these populations, lipid-associated macrophages (LAMs) expand markedly in obese white adipose tissue and exhibit features associated with lipid recognition and uptake, including TREM2 and CD36 [23,24,25]. Their active lysosomal and lipid metabolic programs are closely associated with lipid handling and local tissue remodeling [23,24,26].

Although LAMs have distinct lipid-handling and lysosomal programs [23,24,25,26], it is not known whether TREM2-positive LAMs release EVs at a different rate or carry a reproducibly different miRNA profile.

ATM heterogeneity also extends across adipose depots. Subcutaneous adipose tissue (SAT) and visceral adipose tissue (VAT) differ in their immunometabolic environments, ATM composition, and mechanisms of local macrophage maintenance [17]. SAT- and VAT-derived ATM sEVs have shown contrasting metabolic effects [17], but the ATM features responsible for this difference remain uncertain.

### 2.3. ATM States and Changes in EV-miRNA Cargo

Changes in ATM states may be accompanied by alterations in EV output. In vitro studies have shown that M1/M2 polarization of human primary monocyte-derived macrophages alters both the number of EV particles released and their small non-coding RNA composition [27]. These findings indicate that macrophage states can influence EV release and RNA cargo, although in vitro polarization models do not fully recapitulate ATMs in obese adipose tissue.

Studies of obese ATMs have largely focused on a limited number of candidate miRNAs. Compared with lean mice, EVs derived from obese mouse ATMs contain higher levels of miR-155 [12], while miR-29a and miR-210-3p have also been reported to be increased or enriched [13,14]. Patra et al. [14] further found that lipid-rich and hypoxic conditions increased intracellular miR-210-3p levels in macrophages and its abundance in EV cargo, suggesting that the local microenvironment in obesity may contribute to changes in specific EV-miRNA signals.

Changes in EV-associated miRNA abundance can reflect altered expression in donor cells, selective cargo sorting, or both. Inflammatory stimulation can increase macrophage miR-155 expression, but evidence implicating RNA-binding proteins such as hnRNPA2B1, YBX1, and SYNCRIP in EV-miRNA sorting comes mainly from non-ATM systems [28,29,30,31,32,33]. Direct evidence that these proteins selectively load miR-155, miR-29a, or miR-210-3p into EVs from obese ATMs is lacking.

Ying et al. [12] found no significant difference in total EV production between ATMs from lean and obese mice, whereas Rohm et al. [16] observed increased secretion of small extracellular vesicles (sEVs) after rosiglitazone treatment. Because the studies used different experimental settings and EV measures, their results do not establish a general relationship between ATM state and EV release. In the SAT–VAT comparison, miR-690 expression was higher in SAT ATMs than in VAT ATMs [17]. Functional experiments in the same study implicated miR-690 in the insulin-sensitizing effects of SAT-ATM sEVs; however, its abundance was not directly quantified in the corresponding SAT- and VAT-derived sEV preparations; so, the higher expression in SAT ATMs cannot be taken as evidence of preferential loading into sEVs. Whether these differences define reproducible EV-miRNA profiles across ATM subpopulations remains unresolved.

## 3. Functional Heterogeneity of ATM- and Other Macrophage-Derived EVs

Reported metabolic effects vary with donor source and experimental context: EVs from obese ATMs have been associated with impaired insulin sensitivity, whereas EVs from lean-mouse ATMs, rosiglitazone-remodeled ATMs, selected ATM populations, and some non-ATM macrophage models have shown insulin-sensitizing effects [12,13,14,15,16,17,34,35].

### 3.1. EVs Derived from Obese ATMs and Insulin Resistance

After infusion of EVs from obese mouse ATMs into lean recipients, Ying et al. [12] observed impaired glucose tolerance and systemic insulin resistance. Hyperinsulinemic–euglycemic clamps showed reduced whole-body insulin-stimulated glucose disposal, alongside lower Akt phosphorylation in skeletal muscle. Although miR-155 was enriched in obese ATM-derived EVs and genetic experiments implicated it in systemic metabolic regulation, its specific contribution to the muscle Akt change was not established [12].

Obese ATM-EV studies have also implicated miR-29a and miR-210-3p in insulin resistance, although the recipient-cell models and mechanistic evidence differ between the two candidates [13,14]. Related macrophage–EV studies have reported metabolic effects in non-muscle recipients. EV-associated miR-143-5p from bone marrow-derived macrophages of high-fat-diet-fed mice was linked to altered Akt/GSK signaling and reduced glycogen synthesis in hepatocytes [36]. Under high-glucose conditions, macrophage EV-associated miR-210 was linked to impaired glucose uptake and mitochondrial function in adipocytes [37]. ATM-derived EVs have also been implicated in obesity-associated pancreatic β-cell adaptations [38]. These findings extend the metabolic effects of macrophage-derived EVs to other recipient tissues, without establishing corresponding effects in skeletal muscle.

### 3.2. Insulin-Sensitizing Macrophage-Derived EVs

By contrast, Ying et al. [12] found that EVs from lean-mouse ATMs improved glucose tolerance and insulin sensitivity in obese recipients.

Zhang et al. [34] reported that preparations termed microvesicles from M1-polarized THP-1 macrophages reduced insulin-stimulated Akt phosphorylation, GLUT4 translocation to the plasma membrane, and glucose uptake in human adipocytes; M2-derived preparations had opposite effects. By contrast, EVs from lipopolysaccharide (LPS)-activated macrophages altered inflammation- and metabolism-related gene expression and EV-miRNA composition in adipocytes without significantly changing insulin-dependent glucose uptake [39]. Together, these findings indicate that macrophage polarization alone does not consistently predict the metabolic effects of their EVs.

EVs from IL-4/IL-13-induced M2 bone marrow-derived macrophages (BMDMs) improved glucose tolerance and insulin sensitivity in obese mice, whereas EVs from Dicer-deficient M2-BMDMs failed to reproduce these effects [15]. The RNA reported as miR-690 was enriched in M2-BMDM EVs, and its synthetic mimic increased insulin-stimulated glucose uptake in adipocytes and L6 myotubes [15]. Separately, EVs from IL-4-treated human THP-1 macrophages enhanced insulin-dependent glucose uptake in 3T3-L1 adipocytes and improved glucose tolerance in obese mice, accompanied by reduced hepatic steatosis and inflammation [35].

Rohm et al. [16] found that small extracellular vesicles (sEVs) released by rosiglitazone-remodeled ATMs (Rosi-ATM-sEVs) improved glucose and insulin tolerance in obese mice and increased insulin-stimulated glucose uptake in cultured myotubes. These findings demonstrate insulin-sensitizing activity after pharmacological ATM remodeling but do not establish equivalent effects for EVs from untreated ATMs.

### 3.3. Adipose Depot- and ATM Population-Related Differences in EV Function

ATM-derived sEVs also differ in their metabolic effects across adipose depots and macrophage populations. In L6 myotubes, sEVs from subcutaneous adipose tissue macrophages (SAT-ATMs) of high-fat-diet-fed mice attenuated palmitate-induced impairment of insulin-stimulated glucose uptake, whereas sEVs from the total visceral adipose tissue macrophage (VAT-ATM) population impaired insulin responsiveness [17]. Dicer knockdown in donor ATMs abolished the respective sEV effects, while a miR-690 antagomir attenuated the insulin-sensitizing response to SAT-ATM-sEVs [17].

Within VAT, sEVs from the CD206^+^CD11c^−^ resident-like ATM subset also increased insulin-stimulated glucose uptake in L6 myotubes [17]. Jeelani et al. [17] further linked the preservation of resident ATMs in SAT to CCL26 from adipocytes and adipocyte progenitors. CXCL12 released by resident ATMs was associated with macrophage regulation, while its effects on the production of insulin-sensitizing sEVs were examined using CXCL12-treated BMDMs.

These observations suggest that depot-associated differences in EV metabolic effects should be interpreted in the context of ATM population composition. Table 1 summarizes donor sources, candidate RNAs, recipient models, metabolic outcomes, and evidence context across representative studies.

## 4. Mechanistic Links Between ATM-EV miRNAs and Skeletal Muscle Insulin Sensitivity

Studies of ATM-derived EVs and related experimental models have implicated GLUT4-related glucose uptake and PPARδ-associated metabolic regulation in skeletal muscle insulin responses [13,14]. EV preparations from selected macrophage populations have also shown insulin-sensitizing effects associated with an RNA reported as miR-690 [15,16,17].

### 4.1. EV-miRNAs, IRS1/PI3K/Akt Signaling, GLUT4, and Glucose Uptake

Insulin-stimulated glucose uptake in skeletal muscle largely depends on IRS1/PI3K/Akt signaling and downstream GLUT4 translocation to the plasma membrane mediated by TBC1D4 (AS160). Impairment of this process is a major feature of skeletal muscle insulin resistance [40,41,42,43]. Akt phosphorylation, GLUT4 abundance, GLUT4 translocation, and insulin-stimulated glucose uptake reflect different levels of insulin signaling and metabolic function.

One EV-associated candidate implicated in GLUT4 regulation is miR-210-3p. Patra et al. [14] reported increased miR-210-3p abundance in EVs from obese ATMs, while experiments in C2C12 skeletal muscle cells showed reduced GLUT4 expression following miR-210-3p overexpression. Direct targeting of the GLUT4 3′-UTR was supported by wild-type and mutant luciferase reporter assays in 3T3-L1 adipocytes. Additional in vivo metabolic effects were observed following administration of EVs from experimentally modified BMDMs. These results link miR-210-3p to GLUT4 regulation across distinct models but do not establish a continuous endogenous ATM-EV–skeletal muscle pathway.

In the ATM-EV model, Liu et al. [13] demonstrated the transfer of miR-29a to insulin-responsive cells, including myocytes, although the downstream mechanistic evidence primarily involved PPARδ rather than the IRS1/PI3K/Akt pathway. Evidence linking miR-29 to insulin signaling and GLUT4 regulation comes mainly from independent skeletal muscle and other recipient-cell models. Massart et al. [44] reported elevated miR-29a and miR-29c expression in skeletal muscle from patients with type 2 diabetes mellitus (T2DM). In human primary skeletal muscle cells, miR-29 overexpression reduced IRS1 and PI3K expression and impaired insulin-stimulated glucose uptake, while overexpression in mouse skeletal muscle reduced GLUT4 abundance and glucose uptake [44]. In HepG2 cells, miR-29a targeted the 3′-UTR of the PI3K regulatory subunit p85α and attenuated insulin-induced Akt phosphorylation [45].

### 4.2. miR-29a–PPARδ and Oxidative Metabolism

PPARδ is involved in regulating fatty acid oxidation, substrate utilization, and mitochondrial function in skeletal muscle and is associated with PGC-1α-related oxidative metabolic programs [46,47]. Liu et al. [13] identified PPARδ as a downstream target implicated in the metabolic effects of ATM-EV-associated miR-29a. Inhibition of miR-29a attenuated ATM-EV-induced insulin resistance, whereas the PPARδ agonist GW501516 partially reversed the associated metabolic abnormalities.

Independent skeletal muscle studies have further characterized miR-29a–PPARδ regulation and its metabolic consequences. Zhou et al. [48] observed increased miR-29a expression in skeletal muscle from rats with intrauterine growth restriction (IUGR). In the accompanying experimental work, a luciferase reporter assay supported direct targeting of the PPARδ 3′-UTR by miR-29a. In C2C12 cells, miR-29a overexpression reduced PPARδ and PGC-1α expression, accompanied by lower GLUT4 levels, insulin-stimulated glucose uptake, and ATP production [48]. Under palmitate-induced metabolic stress, miR-29a inhibition improved insulin-stimulated glucose uptake and GLUT4 translocation in C2C12 myotubes, with increased PPARδ expression [49]. In human primary skeletal muscle cells, miR-29 overexpression also reduced fatty acid oxidation and altered PGC-1α expression [44]. These downstream findings were obtained in independent skeletal muscle models rather than the ATM-EV system.

### 4.3. miR-690-Associated Insulin-Sensitizing Effects and Mechanistic Controversies

A different metabolic response has been reported for macrophage EVs associated with an RNA designated miR-690 [15,16,17]. In the M2-BMDM-EV study, Ying et al. [15] proposed Nadk as a downstream target and linked miR-690 manipulation to improved insulin responses. Although miR-690 mimics increased insulin-stimulated glucose uptake in L6 myotubes, functional evidence for Nadk-dependent mediation came mainly from adipocytes and other non-muscle models rather than from L6 glucose-uptake rescue experiments.

Rohm et al. [16] showed that Rosi-ATM-sEVs increased insulin-stimulated glucose uptake in L6 myotubes, an effect blocked by a miR-690 antagomir. In separate L6 experiments involving rosiglitazone-treated BMDM-sEV preparations, changes in miR-690 and Nadk expression were also observed [16]. These results implicate the reported RNA signal in the metabolic response but do not directly establish Nadk-dependent mediation of the Rosi-ATM-sEV effect. Jeelani et al. [17] also implicated this RNA in the insulin-sensitizing activity of SAT-ATM-sEVs without examining Nadk-dependent mechanisms in recipient myotubes.

The molecular interpretation of these findings is complicated by the annotation of mmu-miR-690. Hackenberg et al. [50] questioned whether this sequence meets established criteria for a bona fide miRNA and reported that no corresponding sequence was identified in the human genome. The reported miR-690–Nadk target interaction has not been shown to be conserved in humans. Although macrophage–EV preparations and RNA-directed interventions have shown metabolic effects [15,16,17], these findings do not establish that a bona fide miR-690 mediates these effects through Nadk, particularly in human skeletal muscle.

**Figure 1:**
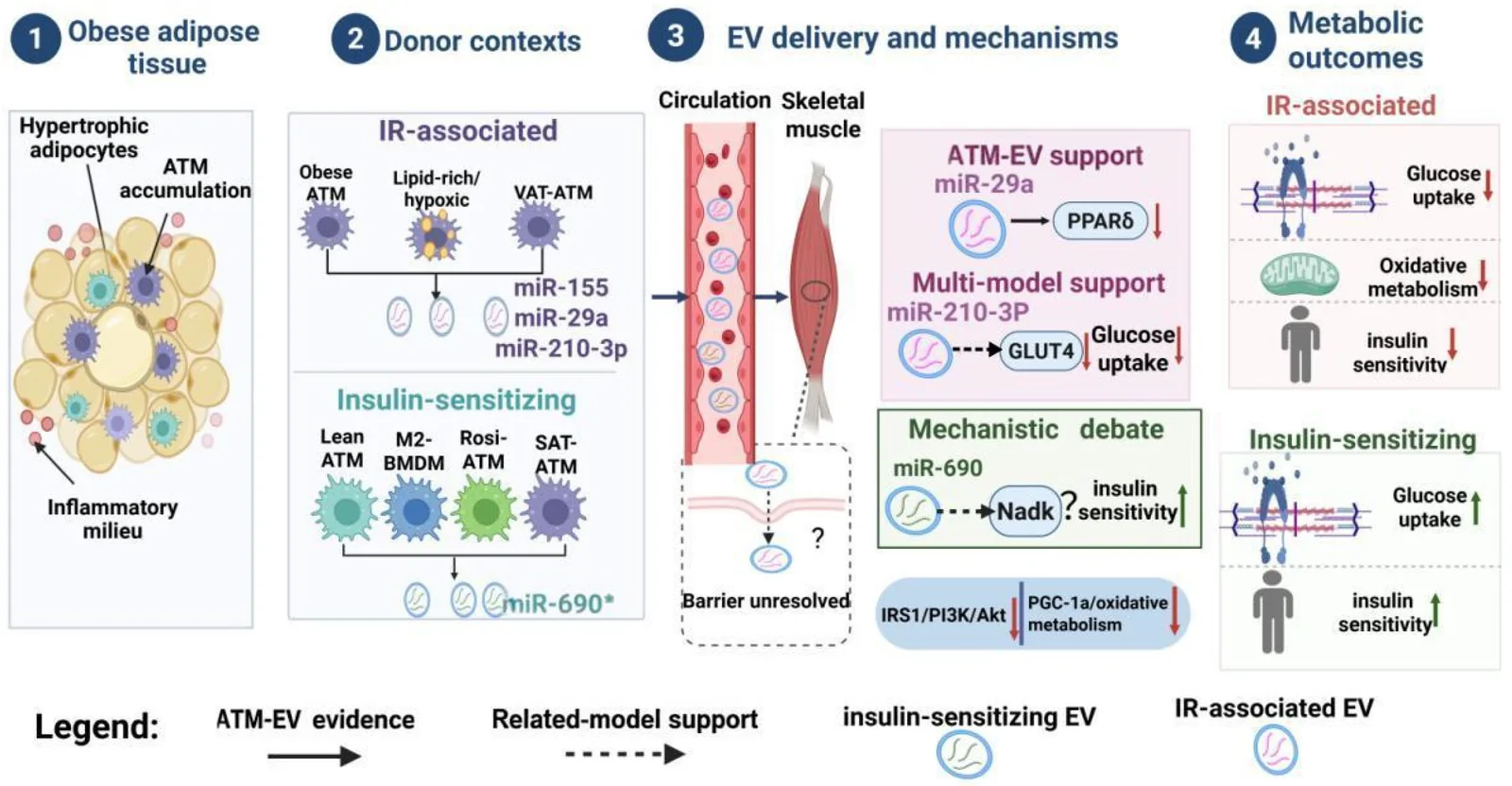
Donor-state-dependent functional heterogeneity of ATM-derived and related macrophage EVs in obesity.

**Figure 2:**
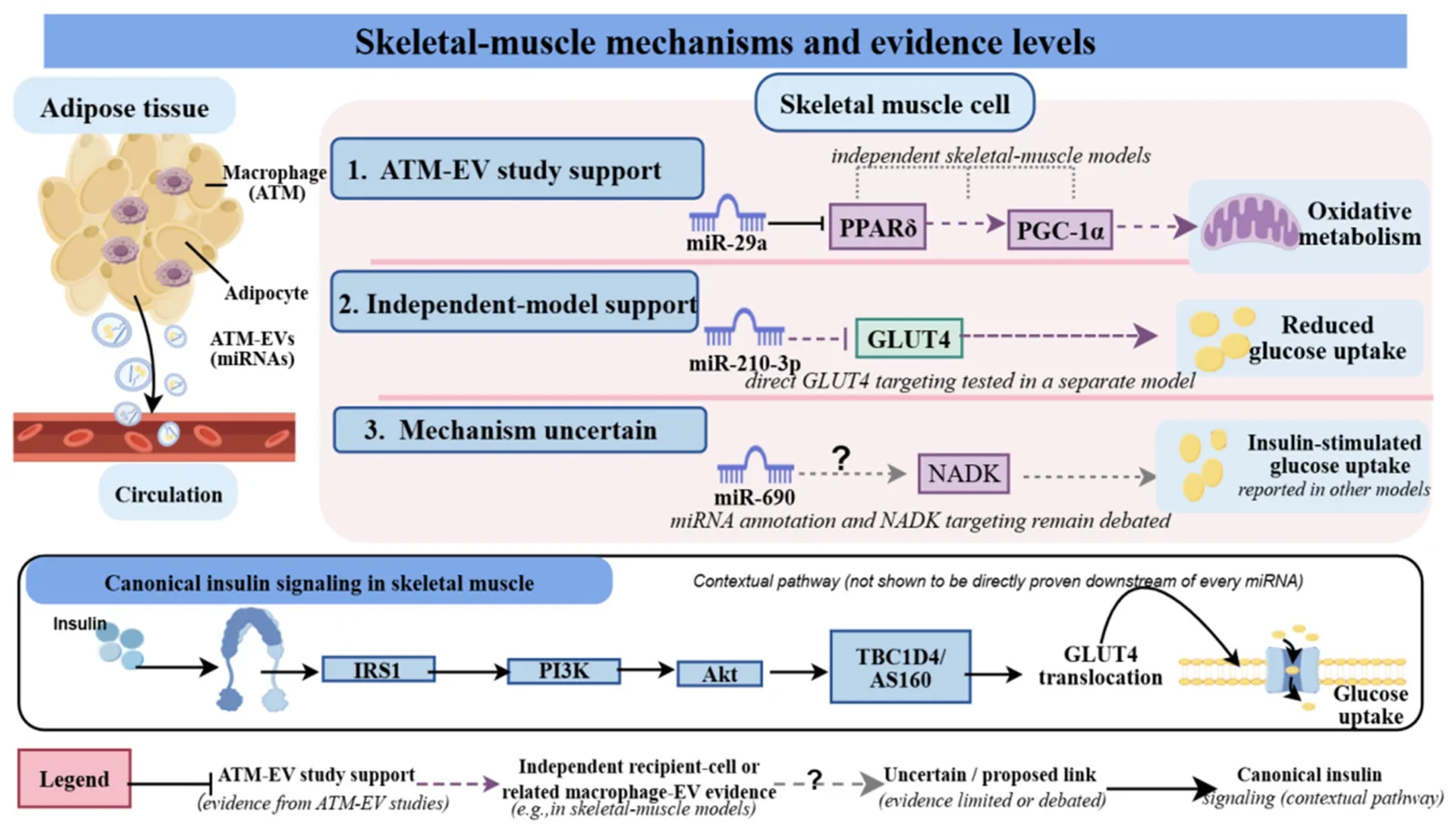
Skeletal-muscle mechanisms and evidence levels for ATM-EV-associated miRNAs.

Taken together, the candidate miRNAs differ in their reported metabolic effects and in the experimental systems supporting their proposed mechanisms. Figure 1 summarizes the donor-state-dependent functional heterogeneity of ATM-derived and related macrophage EVs, whereas Figure 2 focuses on the proposed skeletal-muscle mechanisms and distinguishes evidence from ATM-EV studies, independent recipient-cell models, and unresolved mechanistic links.

The numbered modules depict (1) obesity-associated adipose tissue remodeling, (2) donor contexts associated with insulin resistance or insulin-sensitizing effects, (3) EV-associated miRNA signals and proposed intercellular communication, and (4) reported metabolic outcomes. Pink/purple EV symbols indicate preparations associated with insulin resistance, whereas green symbols indicate preparations associated with insulin-sensitizing effects; downward and upward arrows indicate decreases and increases in the measured metabolic readouts, respectively. Solid arrows indicate relationships supported by ATM-EV studies, whereas dashed arrows indicate findings from related macrophage–EV or independent recipient-cell models. Question marks indicate unresolved or uncertain links. The schematic does not establish endogenous transfer of ATM-derived EVs to skeletal muscle. The miR-29a–PPARδ relationship is supported by ATM-EV studies, whereas the miR-210-3p–GLUT4 relationship draws on different experimental models. The proposed miR-690–Nadk relationship remains uncertain. The asterisk after miR-690 in module 2 is a figure-specific annotation for effects reported in selected M2-BMDM-EV and ATM-sEV models; it does not denote a miRNA star/passenger strand or imply that all insulin-sensitizing EV preparations contain or act through miR-690. In the SAT/VAT comparison, miR-690 abundance was not directly quantified in the corresponding SAT- and VAT-derived sEV preparations. M1/M2 refer to experimentally defined macrophage polarization states and do not fully represent ATM heterogeneity. ATM, adipose tissue macrophage; BMDM, bone marrow-derived macrophage; EV, extracellular vesicle; sEV, small extracellular vesicle; SAT, subcutaneous adipose tissue; VAT, visceral adipose tissue; IR, insulin resistance; Rosi-ATM, rosiglitazone-remodeled ATM; GLUT4, glucose transporter type 4; PPARδ, peroxisome proliferator-activated receptor δ; PGC-1α, peroxisome proliferator-activated receptor gamma coactivator 1 alpha; Nadk, nicotinamide adenine dinucleotide kinase. Created in BioRender. LI, M. (2026) https://BioRender.com/q2htq90.

The canonical insulin-signaling pathway is shown as a contextual framework linking the insulin receptor to IRS1–PI3K–Akt signaling, TBC1D4/AS160 regulation, GLUT4 translocation, and glucose uptake; this framework does not imply that every candidate miRNA directly regulates each component. ATM-EV studies support the involvement of miR-29a in PPARδ-associated metabolic regulation, whereas links to PGC-1α-associated oxidative metabolism are supported by independent skeletal-muscle models. Increased miR-210-3p abundance in obese ATM-EVs is distinguished from GLUT4-targeting findings obtained in separate recipient-cell models. Reported insulin-sensitizing effects associated with miR-690 derive from M2-BMDM-EV and selected ATM-sEV contexts; the annotation of mmu-miR-690 as a bona fide miRNA and its proposed targeting of Nadk remain debated. Solid navy lines denote relationships supported by ATM-EV studies, including exogenous EV-treatment experiments, but do not establish endogenous interorgan transfer or direct molecular binding. Dashed purple lines denote evidence from independent recipient-cell or related macrophage–EV models; dotted gray lines with question marks denote uncertain or disputed links; black arrows denote the canonical insulin-signaling pathway. ATM, adipose tissue macrophage; BMDM, bone marrow-derived macrophage; EV, extracellular vesicle; sEV, small extracellular vesicle; IRS1, insulin receptor substrate 1; PI3K, phosphoinositide 3-kinase; Akt, protein kinase B; TBC1D4/AS160, TBC1 domain family member 4/Akt substrate of 160 kDa; GLUT4, glucose transporter type 4; PPARδ, peroxisome proliferator-activated receptor δ; PGC-1α, peroxisome proliferator-activated receptor gamma coactivator 1 alpha; Nadk, nicotinamide adenine dinucleotide kinase; Rosi-ATM, rosiglitazone-remodeled ATM. Created with Figdraw (https://www.figdraw.com/).

## 5. Physiological Relevance of ATM–Skeletal Muscle EV Communication

Cell and animal studies have shown that EVs derived from ATMs and other macrophages can affect myocyte metabolism and systemic insulin sensitivity [12,13,14,15,16,17]. The physiological relevance of these findings depends on EV distribution to skeletal muscle, the recipient cell types involved, and the functional availability of delivered miRNAs under endogenous conditions.

### 5.1. Endogenous Interorgan Transfer of ATM-Derived EVs

Studies using exogenously administered EVs have provided initial evidence of their distribution to skeletal muscle. Ying et al. [12] detected fluorescence in the liver, adipose tissue, and skeletal muscle following intravenous infusion of fluorescently labeled EVs from lean-mouse ATMs. Similarly, Patra et al. [14] detected fluorescent signals in skeletal muscle and other peripheral tissues after administering labeled EVs from bone marrow-derived macrophages (BMDMs). EV biodistribution depends on the administration route, circulation dynamics, EV characteristics, and tissue-specific uptake [18,51].

However, exogenous EV tracing does not fully reproduce the physiological release and distribution of ATM-EVs in obesity. Lipophilic dyes such as PKH26 may generate non-EV-associated fluorescent particles during labeling, while intravenous administration produces circulating exposure and tissue distribution patterns that differ from those following local EV release from adipose tissue [51,52]. Thus, exogenous tracing provides information on tissue distribution but cannot establish endogenous ATM-EV transfer between adipose tissue and skeletal muscle.

Recent tissue- and cell-specific EV reporter systems have enabled the investigation of endogenous EV release and distribution in vivo. Li et al. [53] developed a Cre-dependent CD63 reporter mouse model for labeling and tracking EVs from specific tissue sources, demonstrating its application to liver- and intestine-derived EVs. Hansen et al. [54] used a Cre-activated CD63-NanoLuc reporter system to monitor adipocyte-derived EVs and observed changes in circulating and peripheral tissue EV-associated signals under high-fat diet conditions.

These reporter approaches have not yet been directly applied to ATM–skeletal muscle communication. The physiological distribution of EVs released by distinct ATM populations therefore remains unresolved. Combining ATM-specific EV labeling with donor-cell-specific manipulation of candidate miRNAs and functional validation in defined skeletal muscle recipient cells could help distinguish tissue distribution, effective intracellular cargo delivery, and metabolic causality. Appropriate controls would also be needed to separate EV-mediated effects from other consequences of donor-cell manipulation.

### 5.2. Skeletal Muscle Recipient Cells and Functional Delivery of EV-miRNAs

Detection of ATM-EV-associated signals in skeletal muscle does not necessarily identify the recipient cell populations. In addition to mature myofibers, skeletal muscle contains vascular endothelial cells, immune cells, and various interstitial cells. Studies of ATM- or macrophage-derived EVs have largely used L6 myotubes, C2C12 cells, and other cultured insulin-responsive cells [13,14,15,16]. These findings indicate that myocytes can respond to EV-associated signals with metabolic changes, but provide limited information about the predominant recipient cell types in intact skeletal muscle.

Even when cultured myocytes respond to macrophage-derived EVs or related miRNA interventions [13,14,16], the amount of EV-associated cargo that reaches skeletal muscle cells at functionally relevant intracellular levels in vivo remains poorly characterized. EV internalization does not necessarily indicate that RNA cargo has reached the cytosol in a form available for gene regulation; uptake can occur without efficient cytosolic cargo delivery [55,56,57,58]. The representative ATM-EV studies also differ in EV isolation and characterization procedures [13,14,16], which complicates direct comparisons of preparation purity, cargo abundance, and the specificity of reported metabolic effects. Because EV preparations may contain non-vesicular co-isolates, metabolic effects cannot be assigned exclusively to EV-associated miRNAs without appropriate EV and non-EV controls and complementary characterization, as emphasized in MISEV2023 [59].

### 5.3. Differences Between Experimental Doses and Endogenous EV-miRNA Levels In Vivo

Most studies of ATM- and other macrophage-derived EVs have used isolated preparations for cultured-cell treatment or exogenous infusion, sometimes alongside miRNA mimics, inhibitors, or donor-cell manipulation [12,13,14,15,16,17]. Experimental doses and reporting metrics vary across studies. Liu et al. [13] administered 30 μg of ATM-derived EV preparations intravenously every 7 days for 2 weeks. Patra et al. [14] administered 2 × 10^6^ BMDM-derived EV particles per mouse at 3-day intervals over 21 days, whereas Rohm et al. [16] administered 1 × 10^9^ Rosi-ATM-sEV particles per mouse twice weekly for 4 weeks. In related in vitro myotube assays, Rohm et al. used 1 × 10^8^ BMDM/ATM-sEVs per well in 24-well plates [16]. These exposures cannot be directly compared because the studies differed in donor source, EV preparation, dose metric, and experimental setting. Rohm et al. estimated that the administered dose corresponded to approximately 10% of the total circulating sEV pool and related it to the rosiglitazone-associated increase in sEVs in HFD mice [16]. This estimate does not establish the endogenous ATM-derived fraction or the amount of functionally available miRNA reaching skeletal muscle.

Studies of selected ATM-EV miRNAs have mainly relied on relative cargo abundance and functional interventions [13,14,16,17]. Quantitative information remains limited regarding candidate miRNA copies per EV, the fraction of circulating EVs derived from ATMs, and the amount of cargo reaching skeletal muscle in a functionally available form. These gaps prevent a direct comparison between experimentally administered doses and endogenous ATM-EV-mediated miRNA exposure.

Quantitative studies in other EV systems provide relevant context. Chevillet et al. [60] quantified miRNAs in various exosome preparations and found that the average abundance of some miRNAs was less than one molecule per vesicle. Although this finding cannot be directly extrapolated to ATM-EVs, it illustrates that the relative enrichment of a particular miRNA does not necessarily indicate its absolute molecular abundance or the amount of functionally available miRNA delivered to recipient cells.

**Figure 3:**
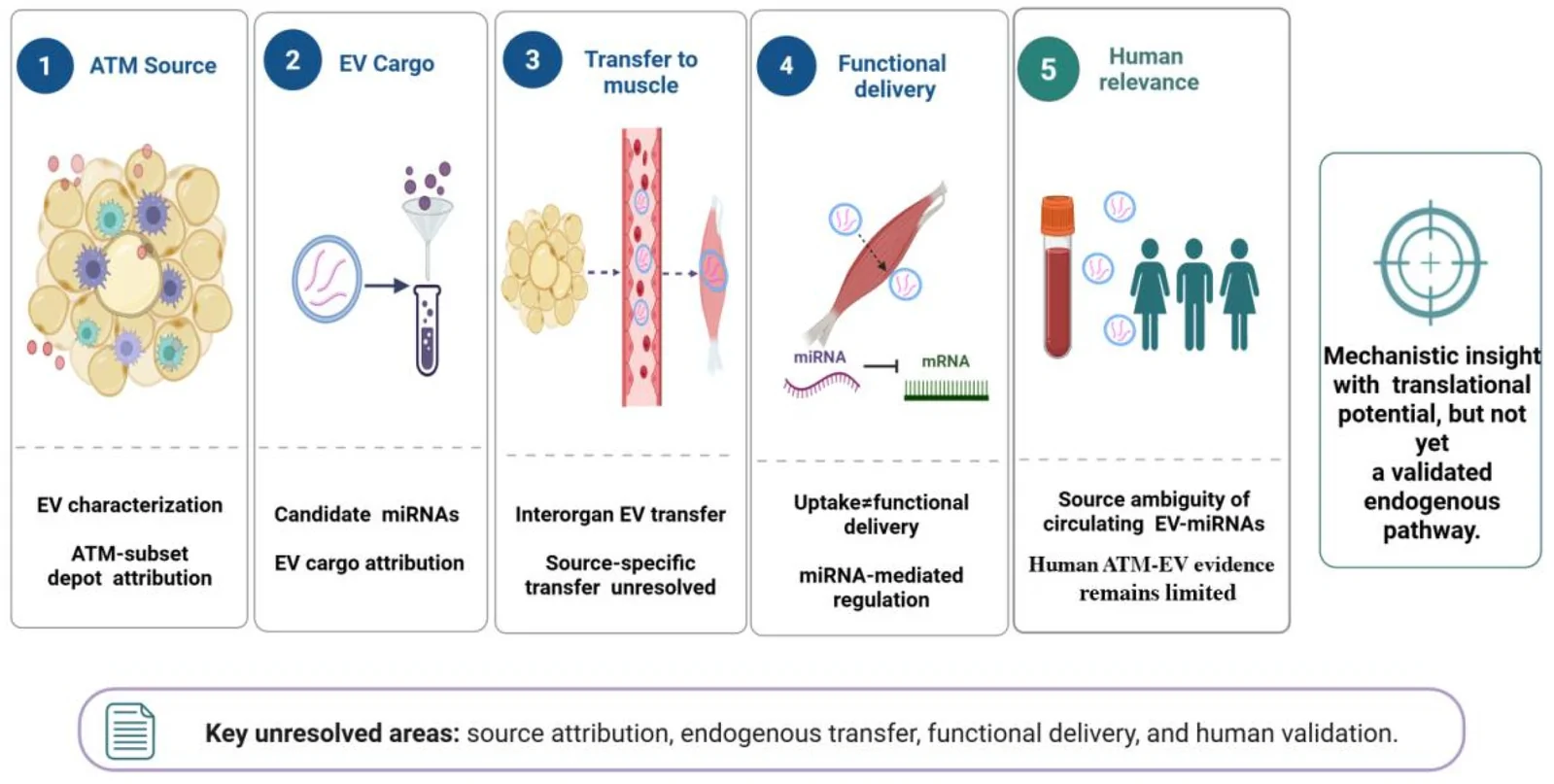
Unresolved steps and translational boundaries in ATM–skeletal muscle EV communication.

Thus, the endogenous ATM-EV exposure required to reproduce these metabolic effects in skeletal muscle remains unknown. Figure 3 summarizes the main unresolved aspects of this communication pathway.

The numbered stages summarize (1) attribution of EVs to ATMs and EV characterization, (2) identification of EV-associated miRNA cargo, (3) endogenous transfer from adipose tissue to skeletal muscle, (4) recipient-cell uptake versus functional cytosolic delivery of miRNA cargo, and (5) evidence for human metabolic relevance. The sequence represents an evidence framework rather than a validated linear pathway. Dashed horizontal separators indicate unresolved evidence boundaries, not established causal transitions. The target icon marks the current status of the field: although mechanistic plausibility exists, an endogenous ATM-EV pathway contributing to skeletal muscle insulin resistance has not yet been validated. ATM, adipose tissue macrophage; EV, extracellular vesicle; miRNA, microRNA; sEV, small extracellular vesicle. Created in BioRender. LI, M. (2026) https://BioRender.com/q2htq90.

## 6. Human Studies and Current Evidence Limitations

Evidence directly establishing an ATM-EV–skeletal muscle pathway in humans remains limited. Available findings derive from human skeletal muscle tissue and primary muscle-cell studies, circulating miRNA and EV analyses, and experimental models using human adipose tissue-related EV preparations or human THP-1-derived macrophages. These evidence types address different questions: human muscle studies support the relevance of candidate miRNAs to muscle metabolism but do not establish an ATM-EV source, whereas circulating miRNA studies rarely resolve tissue of origin or carrier. Some circulating miRNAs are associated with non-vesicular carriers, including Argonaute 2 complexes and high-density lipoproteins [61,62]. Thus, changes in circulating miRNA levels, including those measured in EV-enriched fractions, cannot by themselves establish ATM-EV signaling.

Human adipose tissue-related EV studies provide functional evidence but do not establish ATM-specific signaling. Fuchs et al. [63] reported that plasma- and subcutaneous abdominal adipose tissue-derived EV preparations, termed exosomes in the original study, from participants with obesity and nonalcoholic fatty liver disease reduced insulin-stimulated Akt phosphorylation in human skeletal muscle myotubes compared with preparations from participants with obesity but normal intrahepatic triglyceride content. Mishra et al. [64] found that plasma-derived small EV preparations classified as adipose tissue-derived from individuals with impaired glucose tolerance reduced the expression of several insulin-signaling-related genes in C2C12 myotubes, a mouse cell line, compared with preparations from individuals with normal glucose tolerance. Together, these studies show that human adipose tissue-related EV preparations can alter muscle-cell readouts, but neither identifies ATMs as the donor population nor establishes an ATM-EV contribution to skeletal muscle insulin resistance in vivo.

Among the candidate miRNAs discussed in this review, miR-29 has been relatively well studied in human skeletal muscle. miR-29a and miR-29c expression levels were elevated in skeletal muscle from patients with type 2 diabetes mellitus (T2DM), and studies in human primary skeletal muscle cells also implicated miR-29 in insulin-stimulated glucose uptake and glucose and lipid metabolism [44]. In contrast, clinical associations involving circulating miR-29a have been inconsistent. Some studies reported elevated circulating miR-29a levels in T2DM or positive correlations with the homeostatic model assessment of insulin resistance (HOMA-IR) [65,66], whereas the prospective CORDIOPREV cohort associated lower baseline miR-29a levels with a higher subsequent risk of T2DM [67]. These findings support an association between miR-29 and metabolic status in humans, but circulating levels alone do not establish an ATM origin or skeletal muscle-targeting effects.

Human evidence for miR-155 and miR-210 remains mainly correlational. Mahdavi et al. [68] found no significant difference in serum miR-155 levels between participants with T2DM and non-diabetic controls; within the non-diabetic group, lower levels were associated with obesity and selected metabolic traits. Chen et al. [69] reported higher serum EV-associated miR-210 levels, described as exosome-associated in the original study, in patients with T2DM and a body mass index (BMI) greater than 24 kg/m^2^ than in those with a BMI of 24 kg/m^2^ or lower. Because the latter study did not establish the detected mature miRNA as specifically miR-210-3p, and neither study identified an ATM origin, these findings neither validate nor directly refute the ATM-EV mechanisms reported in mouse models.

Human-cell-line macrophage models provide additional but indirect context. EVs from IL-4-treated THP-1-derived macrophages showed metabolic effects in recipient macrophages and adipocytes and altered metabolic outcomes in obese mouse models [35]. However, this cell-line system does not establish ATM-EV-mediated skeletal muscle effects or reproduce the heterogeneity of resident ATMs in human obesity. A separate line of evidence comes from adipocyte-derived EVs. Sun et al. [70] implicated adipocyte-EV-associated miR-4472 in MEF2D/GLUT4 regulation and reduced glucose uptake in skeletal muscle models. Serum miR-4472 levels in individuals with obesity were elevated and associated with BMI, waist and hip circumference, and fasting glucose [70]. These findings support adipocyte-derived EVs as a relevant non-ATM comparator, but the human serum associations do not establish that circulating miR-4472 is EV-encapsulated or ATM-derived.

miR-690 presents an additional challenge for translation to humans. Its reported insulin-sensitizing effects have been investigated mainly in mouse macrophage–EV and ATM-sEV systems [15,16,17]. However, the annotation of mmu-miR-690 as a bona fide miRNA has been questioned, and its proposed Nadk-targeting relationship remains uncertain [50]. In addition, a clearly corresponding human locus and a conserved target interaction have not been established. The reported mouse findings should therefore be regarded as a model-specific mechanistic lead rather than an established regulatory axis in humans.

Overall, human studies support separate elements of the proposed pathway, including miRNA-related metabolic effects in human skeletal muscle, circulating associations with metabolic traits, and functional effects of human adipose tissue-related EV preparations. However, the available evidence does not establish ATM origin, EV-associated candidate miRNA cargo, endogenous delivery to skeletal muscle, and a resulting metabolic effect within the same human system. Table 2 summarizes the donor sources, skeletal muscle-related findings, reported mechanisms, and human evidence for representative miRNAs while distinguishing ATM-EV findings from related macrophage–EV and non-ATM evidence.

## 7. Therapeutic Implications and Intervention Potential of ATM-EV-miRNA Signaling

The metabolic effects of ATM- and related macrophage-derived EVs vary with donor cell state, suggesting that these signals may be amenable to intervention. Pharmacological and metabolic treatments have been shown to influence macrophage phenotypes, EV release, molecular cargo, and responses in recipient cells. Although direct therapeutic studies targeting ATM–skeletal muscle communication are still lacking, these findings provide a basis for exploring the potential of modulating ATM-EV-miRNA signaling.

### 7.1. Modulation of ATM- and Related Macrophage-Derived EV Signaling by Pharmacological and Metabolic Interventions

The Rosi-ATM-sEV study provides direct preclinical evidence that pharmacological remodeling of ATMs can alter the metabolic effects of their sEVs [16]. In another metabolic intervention model, D-mannose reduced pathological EV release from macrophages under diabetic conditions, accompanied by improvements in hepatic steatosis and insulin resistance. These effects were associated with the regulation of macrophage CD36 function [71], indicating that EV release under pathological conditions can be modified through donor-cell interventions.

Pharmacological interventions can also affect the RNA cargo of EVs. In an in vitro model of lipopolysaccharide (LPS)-stimulated macrophages, semaglutide reduced miR-155 levels in EVs and attenuated their adverse effects on endothelial progenitor cells [72]. As the recipient cells in this model were not skeletal muscle cells, these findings primarily indicate that pharmacological intervention can modify macrophage EV cargo and its effects on recipient cells.

The Rosi-ATM-sEV study directly involved ATMs from obese mice and insulin sensitivity [16], whereas the D-mannose and semaglutide studies demonstrated intervention-associated changes in EV release or miRNA cargo in other experimental models [71,72]. These findings indicate that macrophage EV signaling is amenable to modulation, but do not establish the therapeutic efficacy of targeting ATM–skeletal muscle communication.

### 7.2. Potential Value of Targeting Specific EV-miRNA Mechanisms

Studies manipulating specific miRNAs provide evidence that their associated metabolic effects can be modified, although the sites of action and donor cell sources cannot always be attributed specifically to ATM-derived EVs.

In vivo inhibition of miR-210-3p has shown metabolic benefits in obesity models. Patra et al. [14] reported that local administration of a miR-210-3p inhibitor to visceral adipose tissue in high-fat diet-fed mice increased GLUT4 expression in adipose tissue, skeletal muscle, and liver, accompanied by improvements in glucose tolerance and insulin sensitivity. Another study found that anti-miR-210-3p attenuated adipose tissue inflammation and insulin resistance in obese mice, with effects associated with the SOCS1/NF-κB pathway [73]. However, neither approach specifically targeted ATM-derived EVs, leaving the contribution of ATM-EV-associated miR-210-3p to these metabolic improvements uncertain.

Studies of miR-29a and miR-690 provide additional findings from different experimental contexts. In an ATM-EV model, inhibition of miR-29a or activation of its downstream target PPARδ partially attenuated the associated metabolic abnormalities [13]. In an independent liver model, inhibition of the miR-29 family reduced fasting blood glucose levels and improved certain measures of insulin sensitivity [74]. Exogenous miR-690 mimics also enhanced insulin-stimulated glucose uptake in adipocytes and L6 myotubes [15]. These findings suggest that the respective pathways may be modifiable, although the disputed annotation of miR-690 and its proposed Nadk-targeting relationship limit the mechanistic interpretation of the miR-690 findings [50].

Studies of miR-155 further illustrate how the metabolic effects of a miRNA may vary with its cellular source and delivery context. Although miR-155 in obese ATM-derived EVs has been associated with insulin resistance [12], systemic miR-155 overexpression improved glucose tolerance and insulin sensitivity, accompanied by enhanced insulin signaling in the liver, adipose tissue, and skeletal muscle [75]. These contrasting findings indicate that the effects of a given miRNA should be interpreted in relation to its cellular origin, mode of delivery, and recipient tissue. Based on current evidence, these miRNAs and their associated pathways remain candidate rather than established therapeutic targets.

### 7.3. Potential and Limitations of Engineered EVs as Delivery Platforms for Metabolic Interventions

The metabolic effects associated with ATM-derived EVs have raised interest in whether EV cargo and delivery can be deliberately modified for therapeutic purposes. Although most engineering studies have used EVs from non-ATM sources, they provide experimental evidence for the feasibility of EV-mediated delivery of metabolic regulators. For example, apelin-loaded small extracellular vesicles (sEVs) derived from Wharton’s jelly mesenchymal stem cells (WJ-MSCs) improved glycemic control, glucose tolerance, and insulin sensitivity in mice with type 2 diabetes, accompanied by enhanced Akt/AMPK-related signaling in adipose tissue and adipocytes [76]. EVs with surface-anchored adiponectin also improved glucose tolerance and insulin sensitivity in obese mice, together with enhanced Akt/AMPK signaling in skeletal muscle and liver [77]. These studies demonstrate the potential of engineered EVs in metabolic regulation, although they do not directly address endogenous ATM-EV-miRNA signaling.

Cho et al. [78] developed engineered EVs displaying FGF21 on their membranes and encapsulating miR-223. In a mouse model of metabolic dysfunction-associated steatohepatitis (MASH), these EVs exhibited preferential liver distribution and attenuated hepatic lipid accumulation, inflammation, and fibrosis. Although this study did not involve ATM–skeletal muscle communication, it demonstrated the feasibility of combining protein and miRNA cargo with surface modifications to broaden the delivery options for therapeutic molecules.

The translational maturity of these approaches differs considerably. Pharmacological remodeling of ATMs and administration of Rosi-ATM-sEVs have produced insulin-sensitizing effects in preclinical models [16]. Interventions targeting individual miRNAs remain experimental and are not necessarily specific to ATM-derived EVs [13,14,15,73,74,75]. Engineered EVs from non-ATM sources provide proof-of-concept examples of metabolic cargo delivery [76,77,78], but these have not established efficacy or safety for ATM–skeletal muscle communication. No validated ATM-EV-based treatment for skeletal muscle insulin resistance is currently available.

## 8. Conclusions

Obesity-associated ATM remodeling is accompanied by changes in EV miRNA cargo and metabolic effects. Studies of ATM-derived EVs support the involvement of miR-29a in PPARδ-associated metabolic regulation, whereas the relationship between miR-210-3p, GLUT4, and myocyte glucose uptake is supported by findings from several related experimental models. However, the complete pathway linking ATM-derived EVs to skeletal muscle through miR-210-3p has not been established [13,14]. Studies involving the RNA reported as miR-690 have also shown insulin-sensitizing effects in selected macrophage–EV models, although its annotation as a bona fide miRNA and its proposed Nadk-targeting relationship remain uncertain [15,16,17,50]. These findings suggest a link between differences in ATM and related macrophage backgrounds and EV-associated metabolic effects relevant to skeletal muscle.

Current evidence is derived primarily from cell and animal studies. Exogenous EV treatment has demonstrated effects on skeletal muscle metabolism, while tracing experiments have detected EV-associated signals in skeletal muscle. Nevertheless, the source-specific transfer of endogenous ATM-derived EVs from adipose tissue to skeletal muscle, the principal recipient cell types, physiological exposure levels, and corresponding processes in humans remain insufficiently characterized. Human studies provide complementary findings from skeletal muscle functional analyses, circulating miRNA and EV measurements, and human macrophage models, but direct evidence simultaneously establishing ATM origin, EV cargo, and skeletal muscle functional effects is still lacking.

Pharmacological and metabolic studies have shown that macrophage EV release, miRNA cargo, and functional effects can be modified [16,71,72]. Studies of engineered EVs in other metabolic disease models also provide examples of therapeutic molecule delivery [76,77,78]. Although these advances suggest potential applications for ATM-EV-miRNA research, their therapeutic relevance to ATM–skeletal muscle communication has yet to be established. A clearer understanding of endogenous ATM-EV transfer, physiological exposure, and the corresponding relationships in humans will help assess the contribution of this pathway to obesity-associated insulin resistance and its potential for therapeutic intervention.

## Figures and Tables

**Table 1:** Metabolic and skeletal muscle evidence from ATM- and other macrophage-derived EV studies.

Study EV Source	Candidate RNA/Recipient Model	Main Metabolic Effects	Muscle-Related Findings	Evidence Context
Ying et al., 2017 [12] Lean and obese mouse ATMs	miR-155; mouse recipients and insulin-responsive cells	Obese ATM-EVs impaired systemic insulin sensitivity; lean ATM-EVs improved glucose tolerance and insulin sensitivity in obese recipients.	Muscle Akt phosphorylation was reduced after obese ATM-EV infusion; specific mediation by EV-associated miR-155 was not established.	Direct ATM-EV intervention; systemic and muscle-signaling endpoints differ.
Liu et al., 2019 [13] Obese mouse ATMs	miR-29a; myocytes, adipocytes, hepatocytes, mice	Obese ATM-EVs impaired insulin sensitivity; reducing EV-associated miR-29a attenuated the effect.	ATM-EVs transferred miR-29a to myocytes and impaired insulin responsiveness.	ATM-EV cargo and functional evidence; PPARδ implicated.
Patra et al., 2024 [14] Obese ATMs and modified BMDMs	miR-210-3p; adipocytes, C2C12 cells, mice	Modified BMDM-EVs impaired systemic glucose tolerance and insulin sensitivity.	miR-210-3p reduced GLUT4 in C2C12 cells; direct GLUT4 3′-UTR targeting was tested in adipocytes.	ATM-EV and BMDM-EV evidence; mechanisms tested in separate models.
Ying et al., 2021 [15] IL-4/IL-13-induced M2-BMDMs	RNA reported as miR-690; L6 myotubes, adipocytes, obese mice	M2-BMDM EVs improved systemic insulin sensitivity; Dicer-deficient EVs did not reproduce these benefits.	A miR-690 mimic increased insulin-stimulated glucose uptake in L6 myotubes; Nadk mediation was not established in L6.	Non-ATM BMDM-EV evidence; RNA annotation is discussed in Section 4.3.
Rohm et al., 2024 [16] Rosi-ATMs (sEVs)	RNA reported as miR-690; L6 myotubes, adipocytes, hepatocytes, obese mice	Improved glucose and insulin tolerance in obese mice.	Rosi-ATM-sEVs increased insulin-stimulated glucose uptake in L6 myotubes; a miR-690 antagomir blocked this response.	Direct ATM-sEV functional evidence; Nadk-related results involve additional models.
Jeelani et al., 2025 [17] SAT-, total VAT-, and resident VAT-ATM populations (sEVs)	RNA reported as miR-690 ^†^; L6 myotubes	SAT-ATM-sEVs improved insulin responsiveness, whereas total VAT-ATM-sEVs impaired it.	Resident-like VAT-ATM-sEVs increased L6 glucose uptake; Dicer knockdown abolished depot effects, and miR-690 inhibition attenuated SAT-sEV activity.	Direct ATM-sEV depot/subset comparison; cargo attribution remains incomplete.
Zhang et al., 2015 [34] M1/M2-polarized THP-1 macrophages	No single miRNA prioritized; human adipocytes	M1-derived EVs impaired adipocyte insulin responses; M2-derived EVs had opposite effects.	Not assessed.	Non-ATM THP-1 model.
Phu et al., 2022 [35] IL-4-treated THP-1 macrophages (EVs)	No single RNA prioritized; recipient macrophages, adipocytes, and obese mice.	Reduced cardiometabolic inflammation and improved metabolic outcomes in obese mice.	Not assessed.	Non-ATM human macrophage–EV model.

Note: ATM, adipose tissue macrophage; BMDM, bone marrow-derived macrophage; EV, extracellular vesicle; sEV, small extracellular vesicle; SAT, subcutaneous adipose tissue; VAT, visceral adipose tissue; Rosi-ATM, rosiglitazone-remodeled ATM; GLUT4, glucose transporter type 4; PPARδ, peroxisome proliferator-activated receptor δ; Nadk, NAD^+^ kinase. The general terms EV and sEV are used when vesicle biogenesis has not been established. “Candidate RNA” does not imply direct quantification in every EV preparation or demonstrated causal mediation. † miR-690 was assessed in donor ATMs and functional interventions, but not directly compared in the corresponding SAT- and VAT-derived sEV preparations.

**Table 2:** Donor Sources, Skeletal Muscle-Related Findings, and Human-Related Studies of Selected miRNAs.

miRNA	EV Source/Key Findings	Muscle-Related Findings	Targets/Mechanisms	Human-Related Findings
miR-155	Enriched in obese mouse ATM-EVs [12].	ATM-EV infusion reduced muscle p-Akt; miR-155-specific mediation remains unproven [12].	Muscle-specific target unresolved.	Serum miR-155 did not differ significantly by T2DM status; lower levels were associated with obesity in non-diabetic participants [68].
miR-29a	Enriched in obese ATM-EVs; transfer to myocytes reported [13].	Reduced myocyte insulin responsiveness [13]; independent muscle models showed altered glucose and oxidative metabolism [44,48,49].	PPARδ (ATM-EV model); IRS1/PI3K/Akt, GLUT4, and PGC-1α-related effects (independent models).	Elevated muscle miR-29a/c in T2DM; circulating miR-29a associations were inconsistent [44,65,66,67].
miR-210-3p	Enriched in obese ATM-EVs; modified BMDM-EVs used in vivo [14].	miR-210-3p mimic reduced GLUT4 in C2C12 cells; modified BMDM-EVs reduced muscle GLUT4 in vivo [14].	Direct GLUT4 3′-UTR targeting demonstrated in 3T3-L1 adipocytes [14].	Serum EV-associated miR-210 was associated with metabolic parameters [69] ^a^.
miR-690	M2-BMDM-EVs and Rosi-ATM-sEVs; SAT/VAT ATM-sEV comparisons [15,16,17].	miR-690 mimic increased L6 glucose uptake; antagomir blocked Rosi-ATM-sEV effects [15,16].	Nadk implicated, but muscle-specific mediation and RNA annotation remain uncertain [15,16,50].	No established conserved human counterpart or corresponding Nadk interaction [50].
miR-4472 ^b^	Adipocyte-derived EVs (non-ATM comparator) [70].	Reduced GLUT4 expression and glucose uptake in muscle models [70].	MEF2D/GLUT4 [70].	Serum miR-4472 was associated with obesity-related metabolic measures [70].

Note: ATM, adipose tissue macrophage; BMDM, bone marrow-derived macrophage; EV, extracellular vesicle; sEV, small extracellular vesicle; Rosi-ATM, rosiglitazone-remodeled ATM; SAT/VAT, subcutaneous/visceral adipose tissue; T2DM, type 2 diabetes mellitus; p-Akt, phosphorylated Akt; GLUT4, glucose transporter type 4; PPARδ, peroxisome proliferator-activated receptor δ; PGC-1α, peroxisome proliferator-activated receptor γ coactivator 1α; Nadk, nicotinamide adenine dinucleotide kinase. ^a^ The reported serum EV-associated miR-210 was not established as specifically miR-210-3p or ATM-derived. ^b^ miR-4472 is included as a non-ATM comparator. Its human serum associations do not establish EV encapsulation or ATM origin.

## Data Availability

No new data were created or analyzed in this study. Data sharing is not applicable to this article.
